# Use of information and communication technology and retention of health workers in rural post-war conflict Northern Uganda: findings from a qualitative study

**DOI:** 10.1186/s12911-016-0403-3

**Published:** 2017-01-10

**Authors:** Walter Onen Yagos, Geoffrey Tabo Olok, Emilio Ovuga

**Affiliations:** 1 Department of Library and Information Services, Faculty of Medicine Library Gulu University Gulu, P.O. Box 166, Gulu, Uganda; 2 Department of Computer Science, Faculty of Science Gulu University, P.O. 8 Box 166, Gulu, Uganda; 3 Department of Mental Health, Faculty of Medicine Gulu University Gulu, P.O.Box 10 166, Gulu, Uganda

**Keywords:** ICT usage, Health workers retention, Rural health centres, Post-war conflict, Northern Uganda

## Abstract

**Background:**

Information and communication technologies have become a vital infrastructural asset for use in the retention of rural health workers. However, little is known about the potential influence of ICT use, perceptions of health workers on ICT in healthcare delivery, and contribution of ICT to health care providers’ retention in rural and remote areas in rural post-war and conflict situations of northern Uganda.

**Methods:**

Data from interviews were transcribed, coded and thematically analysed.

**Results:**

Participants generally exhibited low confidence, knowledge and low ICT skills. Majority of participants, however, perceived ICT as beneficial in relation to job performance and health care provider retention in rural areas. Common barriers for the implementation and use of ICT in health centres were inadequate ICT knowledge and skills, poor Internet networks, inadequate computers, inadequate power supply, lack of Internet Modems and expensive access to outside computer centres.

**Conclusions:**

This qualitative study showed low confidence, poor knowledge and skills in ICT usage but positive perceptions about the benefits and contributions of ICT. These findings suggest the need for specific investment in ICT infrastructural development for health care providers in remote rural areas of northern Uganda.

## Background

Information and communication technologies (ICTs) have become a vital infrastructural asset for potential use in the retention of health care providers in remote rural areas [1–5]. Though there have been substantial studies on other factors that influence retention of health workers in rural areas such as pay rise, good housing and others [6], little attention has been put to explore the influence of ICT use on retention of health workers in rural and remote areas [3, 5]. Just as the case in favour of ICT use in promoting the retention of health care providers in rural areas has been made, it is also reasonable to explore the perceptions of health workers on whether ICT facilities and infrastructural development might contribute to retention in rural post-war conflict northern Uganda. ICTs in this study can be defined as tools that facilitate communication, processing and transmission of information by electronic means [7].

Use of information and communication technology is suggested to help break the isolation experienced by rural health workers [8, 9] and it is suggested that health workers are responding to this challenge by adopting e-Health innovation [5]. Benefits of ICT such as online clarification of doubt, opportunity to transmit information, access to training, support to various forms and aspects of decision-making, increased access to specialised tools and access to information may have positive influence on health workers retention in rural and remote areas [3–5]. Reports indicate that specific ICT interventions such as eReferral, teleconsultation, electronic medical record and use of mobile phones have significant benefits to health workers in rural and remote areas [5, 9–12].

Studies however provide us with no definitive understanding of whether the use of ICT and its availability directly influence retention of health workers. Available studies on ICT in health in rural and remote areas have emphasised mainly the benefits of ICT in work processes with little attention to the contributions that ICT might make on the retention of health workers. Bahttacharya and Ramachandran [13] have suggested that ICT is among the factors that can reasonably influence health workers’ intention to stay in urban health facilities in India. Further, training and explaining the benefits of ICT to health workers may also improve retention. However few studies have been done on ICT in health in rural post-conflict situations [5] even though we need more knowledge on other strategies which may influence retention of health workers in underserved areas [3] that are emerging from protracted armed conflict. The post-war conflict situation in this study refers to the situation that followed the devastating and destructive effects of armed conflict in northern Uganda between the Government of Uganda and the Lord’s Resistance Army (LRA), which began in 1986 and ended with the June 2006 peace negotiation [14]. After the war, government priority has been on the reconstruction of what was destroyed and recruiting more health workers to serve in rural post-war conflict northern Uganda. Though these efforts are continuously being implemented, attracting and retaining skilled health workers in rural areas in Uganda still remains elusive. Data on retention of health professionals in Uganda is reported as inadequate; however some of the top strategies that are thought to support retention are: providing accommodation, providing salary top-up for health care providers willing to work in hard-to-reach areas, improving the style of supervision and management, education and training opportunities for health workers, improving work conditions, providing social needs support, and health and access to antiretroviral treatment (ART) [2, 3, 6, 15–18]. Despite all these strategies having been tried, significant shortage of health professionals still remains a big challenge [19, 20], especially having trained health professionals retained in remote rural areas [21, 22]. Within the decentralized healthcare system of Uganda, each of the districts has a well established system of health facilities from level five (district hospitals) at the district headquarters to level one at the village level. Qualified general duty medical officers serve at levels five and four; levels four, three and two being in remote rural areas of each district with little access to social amenities including recreational centres, schools and good road infrastructures. Under the circumstance, health care providers in rural Uganda feel isolated and lack access to peer-support. ICT facilities at these centres may also be rudimentary and may not be functional. Attrition rates from some of the duty stations are often high [23]. Reports in Uganda indicates that only 55%, 45% and 33% posts were filled in HC IV, HC III and HC II in the financial year 2009/2010 respectively resulting in inequitable distribution of health care workers [24, 25].

In Uganda, the unprecedented shortage of health care providers is also attributed to high attrition, low morale and lack of promotional opportunity for health workers. Many times, effectiveness of retention strategies for health professionals is not evaluated and their implementation is done without considering the needs, preferences and perceptions of health professionals themselves [26]. Much as other retention strategies for health professionals have been reported, they do not meet all the requirements for complete attraction and retention [6] and many have yielded little success [27]. The aim of this study was to explore other retention strategies with the potential to enhance retention of health professionals in rural and remote areas. The study explored the perceptions and roles of ICT as a potential factor that might contribute to retention of health workers in rural post-conflict northern Uganda [24].

## Methods

### Study design and sites

The study was conducted in the remote rural areas of six districts in northern Uganda of Adjumani, Amuru, Gulu, Kitgum, Lamwo and Pader, all of which were affected by the armed conflict between Uganda government forced and Joseph Kony’s Lord’s Resistance Army rebels from 1986 to 2006. Participants in this study were stationed at the following health facilities; namely: Atanga HC III, Pajule HC IV, Pader HC III, Pajimo HC III,Palabeck HC III, Madi Opei HC IV, Padibe HC IV, Namokora HC IV, Atiak HC IV, Bobi HC III and Mungula HC IV. The 11 health centres were chosen based on two main criteria: being located in rural post-war northern Uganda and being used as training sites for medical students of Gulu University. This study was conducted as part of a larger ICT needs assessment study for Community Based Education Research and Services (COBERS) sites funded by Medical Education Partnership Initiative – Medical Education for Equitable Services for all Ugandan (MEPI-MESAU) support. COBERS is a practical hands-on medical training program at Gulu University, and it consists of supervised clinical, public health, laboratory and research training at rural health facilities.

### Sampling

Participants from the eleven health centres were purposively sampled. For the purpose of this study, the participants that held in-charge or acting in-charge positions were eligible. Prior telephone calls were made to all the eleven health centres informing them of our study and the purpose of upcoming visits. Participants were informed that the study was voluntary and each of them contacted, willingly accepted to participate in the study and verbal consent was obtained from each of them. We conducted this study from 15th to 21st July 2014. A total of eleven interviews were conducted.

### Data collection

Face-to-face semi-structured interviews using an interview guide specifically designed for the study were conducted by YOW and TOG. Core guiding questions included the following: How do you describe ICT utilization by health workers at the health facility; describe how ICT can motivate you to stay and work in rural health facility; and kindly explain the barriers in the implementation of ICT in this rural health facility. YOW asked questions while TOG took notes during each interview. No audio/audio-visual recordings were made to avoid breach of confidentiality. The interview sessions lasted 30–45 min, with an average of 37.5 min per interview.

### Data analysis

Data collected was transcribed, coded and thematically organised [28]. Qualitative thematic analysis of the interview was done as follows: the replies (notes) of each participant were typed under the corresponding questions in the interview guide (see questions under data collection). The demographic data was converted to quantitative data and a simple frequency table was constructed and reported (See Table 1). Themes arising from the interviews were presented as a direct compilation of the replies including direct quotes from participants (See verbatim quotes in text).

**Table 1:** Characteristics of participants (*N* = 11)

Gender	Frequency
Female	4
Male	7
Position	
Medical Clinical Officer	1
Lab Assistant	1
Nursing Assistant	1
Senior Medical Clinical Officer	2
Health Assistant	1
Medical Officer	3
Enrolled Nurse	1
Nursing Officer	1
Total	11

## Results

### Participants

Eleven face-to-face semi-structured interviews were conducted with eleven health workers categorised in Table 1.

Male participants were disproportionately more than females. The imbalance in gender distribution is a reflection of more male involvement in positions of responsibility in the Ugandan health care delivery system, particularly in remote rural areas [29]. Medical officers and senior medical officers constituted almost half of interviewees. Within the Ugandan health care system, medical officers, senior medical clinical officers, medical clinical officers, nursing officers and laboratory assistants are stationed at Health Centre IV while nursing assistants, health assistants and enrolled nurses may work at both Health Centre IV or III. In this study, 5 of 11 participants were stationed at HC III. Participants at HC V were not included in this study as district hospitals did not qualify as remote rural health facilities.

### ICT utilization by health workers

Eight out of the eleven participants indicated that they sometimes used computers. Of the eight health workers, six health professionals reported being able to use basic internet applications. Many interviewees were able to describe conditions of ICT use by health care providers in the health centres, describing conditions of ICT usage as characterised by little knowledge; some participants reported that they had never used computers and many could not confidently operate computers because of lack of knowledge and skills to do so. The basic knowledge they reported was applied to computer knowledge, especially in Microsoft Word application. Difficulties in using ICT by health workers became clear when all health workers and other government employees were obliged to acquire individual Tax Identification Number (TIN). A health worker in one district narrated what many of them faced due to inadequate computer knowledge.

*“We had challenges when government this year 2014 tasked every employee to get Tax Identification Number (TIN). We had to pay about 10,000 – 20,000 Uganda Shillings to people who are computer and internet literate to create for us e-mails and fill in the online Uganda Revenue Authority (URA) forms.”*

Despite reporting low confidence, knowledge and skills in the use ICTs, many of the interviewees had positive perceptions of the benefits of ICT in supporting health services. When asked about their understanding of the benefits of ICT in health service delivery, interviewees were able to list the following: ICT can help in monitoring surgical operations, accessing knowledge of disease management and drug management, and managing medical records and other medical documents. Participants further reported that keeping data is made easy, tracking information is simplified and access to variety of news, and disease and treatment information through internet is possible with ICTs. Study done by Olok et al [30] in hospitals in northern Uganda indicated that ICT could offer benefits in the delivery of health services. A senior health worker in one district highlighted some of the unique benefits of ICTs as below:

*“I strongly believe that ICT will improve reading culture for the health workers which will improve the quality of health services in rural post-war and post-conflict northern Uganda. Further, ICTs can support learning in the health centre coupled with supporting data work. I am very sure also that internet acts as a source of reference materials which bring creativity among health staff.”*

Generally, health workers in rural post-war and post-conflict northern Uganda exhibited low confidence, knowledge and skills in the use of ICTs. However, health workers have positive perceptions of the benefits of ICT in health service delivery. Regarding the ICT related challenges they faced in ICT use, interviewees were quick to recommend training in ICT and internet skills for all the health workers in rural health centres in northern Uganda to prepare them for any ICT interventions, which may come from government and other health development partners.

### Improving health workers retention through ICT

Information and communication technologies were perceived by interviewees to improve retention in rural health centres in post-war conflict northern Uganda. A senior health worker in one district said: *“ICT is a big boost to health workers motivation”*. One health Assistant in another district said: *“ICT will help motivate many of us to stay at the health centre because ICT, especially internet, makes a global home”*. Since ICT is a resource that can aid the work of health workers, it can be viewed as one factor that influences them to continue working in rural health centres. A senior health worker in a district had the following to say:

*“ICT will keep health workers busy; lack of ICT is the reason we leave the rural areas and move to urban so that socially, we are not disconnected. ICT will keep us together in the profession. Further, ICT brings to us health information resources such as e-books for one to update knowledge, support doctors in self learning and distance learning…we are being kept backward because we always get information last. I am very confident ICT will improve research in medicine at all levels of staffing at the rural health centres in northern Uganda.”*

A Health Assistant in a district explained how ICT would help him stay and work in a rural health facility:

*“I know there is little knowledge about ICTs but I also know that we can use it for entertainment, e-mails, e-learning and linking to other doctors around the country; browse treatments and manuals from other health facilities and medical universities around the world…we will be able to enrol for health management course, which is now a necessity for all health workers in Uganda.”*

It appears clear that ICT introduction at rural health facilities might boost the retention of health workers in rural post-war conflict northern Uganda. Thus on the contribution of ICT to health care provider retention in northern Uganda, a senior health worker in a district said:

*“I am certain computers and internet can motivate doctors to stay at one place because they will be sure to get information, leisure, entertainment and so on…staff mobility can reduce, I think by up to 60% … our stay will become easier and simpler especially when patients are not around; and also treatment of some sicknesses can be aided by access to the Internet. When computers and internet are available on site, then there will be no unnecessary movement because we will be in a global interconnected village through ICT.”*

One health centre actually had tried an innovative way to keep their staff at the health facility through the use of television. A senior health worker in the health facility concerned explained how she tried the use of digital satellite television (Dstv) to keep staff at the health centre and had this to say:

*“For me, ICT is a very viable way for staff retention. Here, almost all the weekend health staff travel to town leaving only those on call at the centre. As a trial, I decided to install Dstv at the centre, which has helped me to keep almost all staff at the health centre. I expect availability of computers and internet to even do much better on staff retention than my Dstv.”*

### Barriers to the implementation of ICT in rural health centres

Though ICT appears to contribute to the retention of health care providers in rural areas, many challenges might slow down it use, such as inadequate ICT knowledge and skills of health workers; poor internet connectivity; inadequate computers; inadequate electricity supply; lack of internet Modems; expensive access to outside computer centres; inadequate space for ICT; lack of ICT policy and inadequate ICT officers. For example a health worker in one district had this to say:

*“Many of our staff can not confidently operate computers and they lack information, knowledge and skills to do so…the staff cannot also use computer applications and they need training.”*

For internet access for example, health workers had to travel long distances to look for places with strong internet connectivity. A senior medical officer narrated this on poor internet network:

*“Internet is not available at the health centre and not even at the trading centre. I think we truly have problems going to the district headquarter to look for internet. Whenever we go to the town about 47 km from the health centre, we travel together with medical students to access internet in town. The frequency of our travels is every after two days”. It is very difficult to predict how long this method can keep us going. All network services around are very poor although we have some kind of access to MTN and Airtel networks.”*

Similar views were shared by a health worker in one of the remotest districts in northern Uganda.

*“I think there is really urgent need to install ICT in this health centre. Imagine we are forced to travel 49 km to town just to do photocopying. Access to ICT facilities is very poor in this district in general and the only option now is to go to a town in a neighbouring district.”*

Some health centres visited have computers, though inadequate and some in poor working conditions. Five health centres out of the 11 visited had no computers. A health worker in a district had this to say:

*“For Health workers in this health centre, I do not think if any of us has ever used computers and internet. We have no computers here and health workers are not at all exposed to computer usage. We know the benefits of ICT but we can only turn these benefits to health services when computers are available and all of us know how to operate it. I can tell you that there is only one place here at a sub-county office where people access computer and internet services, but the ICT facility is not reliable and it is not accessible to all health workers.”*

Inadequate electricity supply also presents challenges to ICT implementation, as most rural areas in northern Uganda are not electrified. After the armed conflict in northern Uganda, many health centres received solar system as the main power supply. A senior health worker in a district explained:

*“The main source of power supply at health centres after the war was solar. We are still continuing to use solar but many of the batteries have expired. The government main grid you see here is not yet functioning but electricity service poles are being installed. Some solar systems which we have are broken down. We currently run a generator which is again very expensive to sustain because of the expensive fuel.”*

Apart from the challenges that impede the availability and use of ICT facilities, the theft of existing infrastructures apparently affects rural health facilities in northern Uganda. A health worker said:

*“This health centre had four new computers but unfortunately two were stolen before the gate was constructed. I can tell you that there is rampant stealing of computers from health centres. Just two week ago, one computer was reported stolen from a nearby health centre.”*

## Discussion

Following the ICT revolution, information and communication technologies appear to have become a vital infrastructural asset in health and in the retention of rural health care providers. Using qualitative study we explored the experiences and perceptions of health workers about their ICT use, benefits of ICT in health services, contribution of ICT to Health workers retention, and barriers to ICT implementation in rural post-war and post-conflict northern Uganda.

Some participants reported low confidence, knowledge and skills to operate ICTs but reported positive perceptions on the benefits of ICT among health workers. What probably exists among health care providers in rural health facilities is the very basic computer knowledge, especially in Microsoft Word and internet applications. Our findings in the present study do not corroborate the findings in a study by Woodward and colleagues [5] in which health workers reported a range of engagement activities with ICTs. Woodward and colleagues [5] carried out their study in post-conflict situations targeting mainly health workers already experienced in ICT applications and usage. The unique contribution of our study is that we interviewed directly health workers engaged in a rural post-war and post-conflict situation where ICT applications are hard to comeby in health service delivery. The positive perceptions of ICT in this study however corroborate the study of Woodward and colleagues’ study [5] who found that health workers had positive perceptions of e-health. These results especially mean that there is urgent need to invest in ICT in rural post-conflict situations, create awareness of ICT and expose health workers to ICT and train them to use ICT in health service delivery. The positive perceptions of ICTs can be understood to indicate that health workers are willing to embrace ICTs in rural post-war northern Uganda.

Findings of the study indicate that health workers perceived ICT availability and use to contribute to their retention in rural areas. Many studies have reported the contribution of ICT in retention of health workers [1, 3, 5, 8] while other studies have reported the benefits of ICT in health care work processes [5, 11, 12, 31]. In northern Uganda many health workers’ retention strategies do not emphasise the role of ICT in addressing the needs and interests of health care providers but rather rely on intrinsic and extrinsic motivation (traditional) strategies of retention [2, 3, 15–18]. Our finding that ICT improves retention of health workers in rural situations is well placed. The results of our study appear to indicate that investing in health centres’ ICT might prove to be one very important avenue for boosting retention of health workers in rural areas. In order for ICT to influence retention positively, there is need to train health workers [32]. Further, increasing the presence of ICT in rural health centres will likely improve retention and availability of health workers in rural areas including those in rural post-war conflict situations.

Despite the other findings from this study, there are several barriers which appear to impede the implementation and use of ICTs in rural northern Uganda. These were inadequate ICT knowledge and skills of health workers; poor Internet services/networks; inadequate computers; inadequate power supply; expensive access to outside computer services from trading centres; inadequate space for ICT; lack of ICT policy and ICT officers. These barriers are similar to the findings of Woodward et al [5] in which poor, unreliable and unaffordable internet was reported. These current barriers need to be addressed directly by making ICT intervention one of the priority strategies in the plan for the reconstruction of post-war conflict situations. Massive training and investment in ICT facilities by government and other development partners based on the needs of health workers should be considered as a priority [32]. If ICT is to improve health workers’ retention as aforementioned, then the elimination of the identified barriers should be one of the immediate steps to take in encouraging or fostering retention of health workers in rural areas. We wish, however, to point out that our participants equated ICTs mainly with computers and internet which were mainly used for word processing, data entry and communication purposes. There is now a range of ICT equipment other than desktop computers, all of which could be provided to rural health care providers. For example, several varieties of smartphones are now available on the Ugandan market that could as well help health care providers in rural areas keep in touch with professional colleagues, relatives, and friends, and to communicate and access health information right from their rural stations. In the training of health care providers, the diversification of available ICT infrastructures might need to be brought to the attention of health care providers.

### Study limitations

Participants for the study were purposively sampled, selecting only the health centre in charges and those left in acting capacity. The study was also only based on interviewee perceptions. The leadership of health centres may not adequately be in position to address issues in regard to ICT interventions and retention as seen by other rural health facility staff. Further, our study did not adequately address activities considered when using ICT and our participants, by virtue of their management positions, might have been more exposed to the values and uses of ICT facilities. In this respect it might be considered that our results may be biased and skewed. However we believe that the information that our participants provided was based on wide experience based on their day-to-day interactions with other health facility staff. The information we have reported can therefore be regarded as a true reflection of the real-time situation of ICT facility availability, utilization and values in rural health facilities in post-war conflict northern Uganda. The strength of our study is that all participants were interviewed in their work places (health Centres) and were able to give exclusively what they felt and knew about ICT in rural northern Uganda. In addition, the interviews were done in eleven health centres spread over six districts in northern Uganda; the sample is geographically and reasonably representative of northern Uganda in terms of area coverage. Future studies might use a larger population of health workers, combining qualitative and quantitative approaches to study the use of ICT in improving population health in rural areas of Uganda. Despite its identified limitations, this study provided insight in to the perceptions of health workers about their ICT experiences, benefits of ICT in health services delivery, contribution of ICT to Health workers retention, and barriers to ICT implementations in rural situations in northern Uganda.

## Conclusions

This study explored the role of ICT in health care provider retention in rural northern Uganda. We identified low confidence, knowledge and skills to operate ICT among health workers as important factors to consider in promoting retention strategies in rural areas of Uganda. Positive health care provider perceptions of the benefits of ICT and its contributions to health workers’ retention might facilitate retention of rural health workers. To improve health care provider retention in rural areas it might require investment in ICT infrastructural development along with offering training opportunities for most health care providers.
